# Leber's hereditary optic neuropathy like disease in *MT-ATP6* variant m.8969G>A

**DOI:** 10.1016/j.ajoc.2024.102070

**Published:** 2024-05-03

**Authors:** Cansu de Muijnck, Mary J. van Schooneveld, Astrid S. Plomp, Richard J. Rodenburg, Maria M. van Genderen, Camiel J.F. Boon

**Affiliations:** a Department of Ophthalmology, University Medical Center Utrecht, Utrecht, the Netherlands; b Department of Ophthalmology, Amsterdam University Medical Centers, Amsterdam, the Netherlands; c Department of Human Genetics, Amsterdam University Medical Centers, Amsterdam, the Netherlands; d Amsterdam Reproduction and Development Research Institute, Amsterdam, the Netherlands; e Radboud Center for Mitochondrial Medicine, Departments of Pediatrics and Genetics, Radboud University Medical Center, Nijmegen, the Netherlands; f Bartiméus Diagnostic Center for Complex Visual Disorders, Zeist, the Netherlands; g Department of Ophthalmology, Leiden University Medical Center, Leiden, the Netherlands

**Keywords:** MT-ATP6, Leber's hereditary optic neuropathy, Optic atrophy

## Abstract

**Purpose:**

To describe a case with Leber's hereditary optic neuropathy (LHON) like optic atrophy in the presence of *MT-ATP6* gene variant m.8969G > A.

**Observations:**

A 20-year-old patient with a history of mild developmental delay, mild cognitive impairment, and positional tremor presented with subacute painless visual loss over a few weeks. Mitochondrial genome sequencing revealed a variant in *MT-ATP6*, m.8969G > A (p.Ser148Asn). This variant was previously reported in association with mitochondrial myopathy, lactic acidosis, and sideroblastic anemia (MLASA) and with nephropathy, followed by brain atrophy, muscle weakness and arrhythmias, but not with optic atrophy.

**Conclusions and importance:**

Rare variants in *MT-ATP6* can also cause LHON like optic atrophy. It is important to perform further genetic analysis of mitochondrial DNA in genetically unsolved cases suspected of Leber's hereditary optic neuropathy to confirm the clinical diagnosis.

## 1 Introduction

Leber's hereditary optic neuropathy (LHON) is characterized by painless, sequential bilateral acute vision loss, mostly in the second or third decade of life due to mitochondrial genomic mutations. Although some LHON patients may show some spontaneous recovery of visual acuity, most patients sustain severe visual loss. Optic atrophy is the main manifestation of LHON but some patients also suffer from cardiac arrhythmias, myopathy, or neurological manifestations such as dystonia and peripheral neuropathy.^1^ Next to mitochondrial mutations, recently some autosomal recessive variants causing LHON also have been described.^2^

Ninety percent of the patients with LHON harbor one of the three variants (m.3460G > A, m.11778G > A, and m.14484T > C) in the mitochondrial genes *ND1, ND4,* and *ND6,* respectively. In the remaining 10 %, variants in some nuclear (*MCAT, DNAJC30, NDUFA12*) and seven other mitochondrial genes have been described including *MT-ATP6*.^2^, ^3^, ^4^, ^5^ *MT-ATP6* is a mitochondrial gene encoding the F_0a_ subunit of complex V in the electron transport chain. Complex V - or ATP synthase - is responsible for the formation of ATP from ADP at the last step of oxidative phosphorylation. Variants causing complex V insufficiency have been associated with various diseases, including Leigh syndrome, NARP (neuropathy, ataxia and retinitis pigmentosa) and mitochondrial myopathy, lactic acidosis, and sideroblastic anemia (MLASA).^6^

*MT-ATP6* variants are thus far reported in a few studies in association with LHON-like clinical pictures.^7^, ^8^, ^9^ Although these studies show some mitochondrial dysfunction in the presence of *MT-ATP6* variants, establishing causality between *MT-ATP6* variants and LHON remains challenging due to the limited number of studies, high homoplasmy rates in unaffected individuals in these studies, and complex protein dynamics of ATP synthase complicating the assessment of functional consequences of *MT-ATP6* variants.

In this paper, we present a 31-year-old man with mild developmental delay, mild cognitive impairment, positional tremor, optic atrophy with typical LHON onset, and bilateral retinal detachments. Mitochondrial DNA sequencing revealed a heteroplasmic variant in *MT-ATP6* (m.8969G > A) while array CGH showed a normal array profile and sequencing of *OPA1, SPG7* and *PLP1* revealed no pathogenic variants. The pathogenicity of the m.8969G > A variant was confirmed via segregation analysis and functional assays.

## 2 Case report

The proband is a 31-year-old man, born at term with forceps delivery following a normal pregnancy, with a birth weight around 4000 g. Postnatal there were no complications. The parents are not consanguineous. He has a younger sister, who is healthy. No other family members were known to have optic atrophy and/or mitochondrial disease.

In the first two years of life, he had frequent visits to the youth and family center because of delayed psychomotor development. His gross and fine motor skills developed at later ages than expected, he was clumsy, and psychological tests showed younger intellectual capacity than expected from the calendar age. After various early intervention service trajectories with different diagnoses (attention deficit and hyperactivity disorder, non-verbal learning disabilities, and low intellect), he was referred to a pediatric neurologist at a tertiary center at the age of fifteen. Anamnestic he had migraine type headaches. On physical examination, he had mild intention tremor, a length difference between his lower extremities, and a positive Babinski sign at the left side. He had minimal anisocoria (L > R) and convergence weakness of the left eye. Cerebral MRI was normal. He received the diagnosis of cerebral palsy and he was referred back to early intervention services. The patient complained sometimes of tachycardia. At the age of 23 he went to a cardiologist, who did not find any abnormalities.

**Fig. 1:**
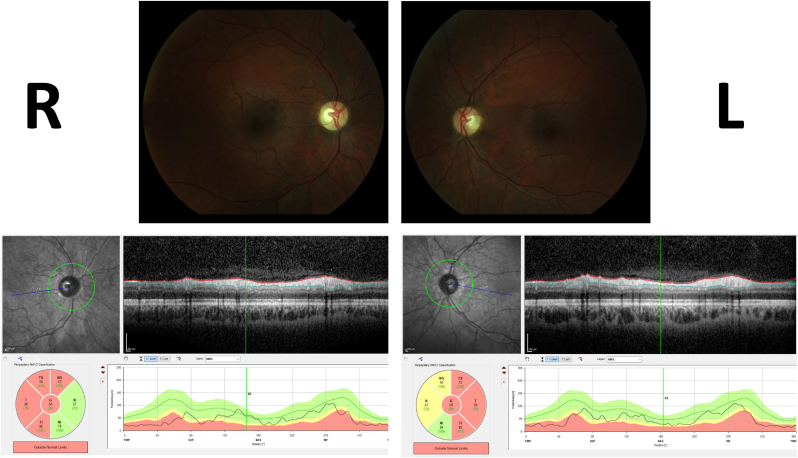
Fundoscopy and optical coherence tomography (OCT) imaging of the optic nerve. Pale optic discs were seen on fundoscopy and retinal nerve fiber layer was attenuated bilaterally on the OCT.

At the age of 20, he experienced a subacute, painless loss of vision that progressed in a couple of weeks in both eyes. Visual complaints were widespread in the whole visual field. On ophthalmological examination, he had a best-corrected visual acuity of 20/200 Snellen in both eyes with no visible abnormalities on fundus imaging. In spite of the decreased visual acuity, he made a visually well-oriented impression. Automated perimetry was not reliable due to high false positives. Goldmann kinetic perimetry showed a relatively preserved peripheral visual field in both eyes. Visual evoked potentials showed no recordable responses. A repeated MRI of the brain showed no pathological process. Fluorescein fundus angiography showed no papillary leakage. At follow up six months later, patient had developed pale optic discs on ophthalmoscopy, and visual acuity had worsened to 1/60 (Fig. 1). On optical coherence tomography (OCT), retinal nerve fiber layers (RNFL) were attenuated, especially in the temporal and superior quadrants (Fig. 1).

**Fig. 2:**
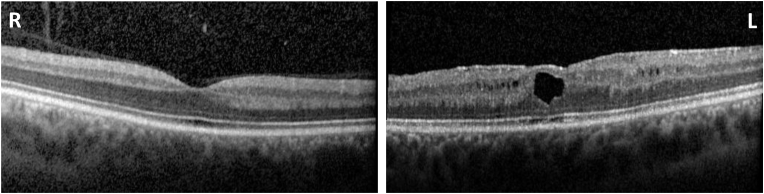
Optical coherence tomography scan of the macula from the last follow up (30y). On the right eye, retinal nerve fiber layer is markedly attenuated. On the left eye, there are few intraretinal fluid collections.

One year after the subacute vision loss, the patient presented with complaints of a blurry spot in the left eye with distortions of the image after a plane trip. Fundus examination revealed a local retinal detachment inferiorly in the right eye and an extensive retinal detachment including the macula in the left eye. Visual acuity was still 1/60. Both eyes were treated operatively. The visual acuity slightly improved to 2/60 after the surgery. Four years after the vitrectomy, central macular edema developed in the left eye that was more widespread than one would expect from microcytic edema of LHON. This edema was treated medically without a clear benefit for visual acuity. At the last follow up visit, he had few mild intraretinal fluid collections on the macular OCT of the left eye and stable a best corrected visual acuity of 20/400 Snellen in both eyes (Fig. 2).

Genetic testing of mitochondrial DNA with suspicion of LHON revealed the m.8969G > A (p.Ser148Asn) variant in *MT-ATP6* (GeneChip ® Human Mitochondrial Resequencing Array 2.0, Affymetrix Inc.), which was initially classified as variant of unknown significance. The variant was validated with Sanger sequencing. Sequencing of *OPA1* for the differential diagnosis of dominant optic atrophy and *SPG7* & *PLP1* for hereditary spastic paraplegia revealed no pathogenic variants. Array CGH designed to detect copy number variations associated with intellectual disability showed no chromosomal anomalies (Agilent 180K oligo-array, Amadid 023363; Agilent Technologies). Genetic testing and the functional analysis were performed in the Translational Metabolic Laboratory of the Radboud University Medical Center and the Genome Diagnostic Laboratory of Amsterdam University Medical Centra (both ISO15189 accredited but not CLIA certified).

**Fig. 3:**
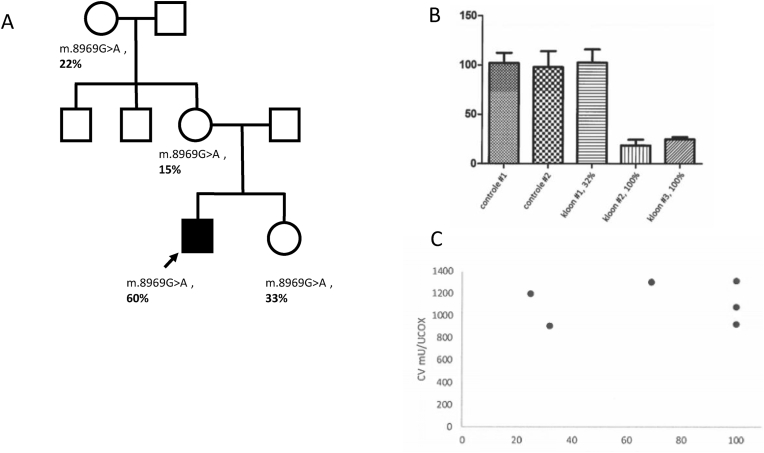
**A.** Family tree, showing the segregation analysis and heteroplasmic state (%) in blood of different family members. Sister, mother and the maternal grandmother of the patient had the same variant with a heteroplasmic state of 33 %, 15 % and 22 %, respectively. **B.** Results of mitochondrial respiration rate analysis via seahorse respirometer among cell clones with different heteroplasmy levels showing decreased oxygen consumption levels in homoplasmic clones compared to the heteroplasmic clone and two healthy controls. **C.** Levels of complex V in cell clones with different heteroplasmy levels. Complex V levels varied and were not consistently low in homoplasmic clones.

Muscle and skin biopsies were performed to assess the pathogenicity of the m.8969G > A (p.Ser148Asn) variant in *MT-ATP6*. The patient harbored the variant with variable heteroplasmy levels in different tissues: 93 % in muscle, 69 % in fibroblasts, and 60 % in blood (Ion Torrent PGM). Histological examination of the muscle cells revealed nonspecific myopathy signs with fiber atrophy and fat accumulation. On electron microscopy, unusually high numbers of mitochondria were observed between the Z bands with normal morphology. The initial muscle biopsy did not reveal reduced levels of respiratory chain enzymes, but the results were inconclusive as the complex V could not be measured in muscle cells due to the unsuitability of the frozen biopted tissue for this particular test. Cultured fibroblasts from the skin biopsy showed neither respiratory enzyme levels reduction nor decreased respiration levels on Seahorse respirometer. Considering the high heteroplasmy levels in previously reported studies on *MT-ATP6* related disease, we thought these results might be due to the relatively low heteroplasmy (69 %) in the patient's fibroblasts and we were curious whether cell clones with higher heteroplasmy would display a different mitochondrial performance. We isolated homoplastic cells via single cell sorting technique and performed the same tests. Compared to healthy controls and the clones with lower heteroplasmy, homoplasmic cell clones exhibited significantly decreased mitochondrial respiration capacity. We could not find a correlation between the complex V levels and the decreased mitochondrial respiration capacity (Fig. 3).

Segregation analysis showed that the sister, mother and the maternal grandmother of the patient had the same variant with a heteroplasmic state of 33 %, 15 % and 22 %, respectively (Fig. 3). Together with this information and the mitochondrial respiration analysis, we concluded that the variant is probably the cause of the phenotype of this patient.

After genetic diagnosis, the patient was referred to Radboud Center for Mitochondrial Medicine for systemic assessment for mitochondrial disease. During this visit, he had an ECG with mild signs of left ventricular hypertrophy which did not fulfill the diagnostic criteria completely and negative T tops. Serum organic acids and homocysteine measurements showed slightly elevated lactate levels, abnormally high alanine with distorted alanine/lysine ratio. All together these findings can be in line with suboptimal mitochondrial function.

## 3 Discussion

In this study, we describe a patient with LHON like clinical presentation in the presence of *MT-ATP6* variant m.8969G > A (p.Ser148Asn). This variant was previously described in the literature in association with severe phenotypes such as mitochondrial myopathy, lactic acidosis, and sideroblastic anemia (MLASA); nephropathy followed by brain atrophy, muscle weakness and arrhythmias, and milder phenotypes with lactic acidosis and intellectual disability.^10^, ^11^, ^12^, ^13^ After conducting a literature review utilizing PubMed, Google Scholar, and Embase, we did not encounter another study linking this variant to LHON like disease before.

Data on the association of *MT-ATP6* with LHON is limited. Lamminen et al. described a proband with an *MT-ATP6* variant and LHON like clinical picture.^7^ Although the reported variant (m.9101T > C) was in homoplasmic state along with the proband in 6 other unaffected family members, authors of this study showed the pathogenicity of this variant in a follow-up study.^14^ In cybrid cell lines, cells fused with enucleated patient cells with the m.9101T > C variant exhibited decreased oxidative phosphorylation efficiency. In another study, Lopez et al. reported a 38-year-old patient with a history of visual loss since 4 years, diagnosed as atypical LHON.^8^ This patient had the m.9029A > G variant in blood cells, and functional studies with hybrid cells showed decreased endogenous oxygen consumption. Two other *MT-ATP6* variants (m.3483G > A and m.9011C > T) have been reported by Shidara et al. as potential new LHON-causing variants, but this study did not include a functional assessment.^9^ Although the number of cases reported with *MT-ATP6* variants and LHON is limited, together with the findings of this study, variants in *MT-ATP6* seem to be a rare cause of LHON.

Retinal detachments are not typical disease manifestations for inherited optic neuropathies. Although few studies report patients with inherited optic neuropathy developing retinal detachments, these seem rather coincidental findings than an actual part of the phenotype.^15^, ^16^, ^17^ Our patient had no risk factors for retinal detachment. The etiology of the bilateral retinal detachment in this young male without myopia remains a mystery.

Assessing mitochondrial variants can be challenging due to a number of characteristics of the mitochondrial genome. In individual cells, mutated mitochondrial alleles generally coexist with wild-type alleles in different proportions, which leads to a situation called heteroplasmy. Different tissues in the same individual may show different levels of heteroplasmy. In general, heteroplasmy levels above 60%–70 % are considered necessary to overcome the function of the normal alleles and elicit phenotypic changes, although this may vary for some mtDNA variants. Moreover, incomplete penetrance is a known phenomenon in LHON, even in homoplasmic families carrying one of the three most common variants.^18^ In our patient, skin fibroblasts showed a heteroplasmy level of 69 % and initial functional assessments with these cells showed no decreased mitochondrial function. We isolated cells homoplasmic for m.8969G > A via single cell sorting to observe the functional outcomes of the variant in the absence of compensation by the wild type alleles. Cells with 100 % mutated *MT-ATP6* showed diminished mitochondrial respiration levels. Previous studies in the literature reporting *MT-ATP6* variants (including m.8969G > A) also describe heteroplasmy levels above 80 % in affected individuals.^6^ Therefore, heteroplasmy levels exceeding 90 % may be needed to detect functional anomalies in *MT-ATP6* variants in patient-derived cell cultures. Segregation pattern in this family and the heteroplasmy levels in the different tissues of the patient were also in line with previously described features of heteroplasmic mitochondrial disease, supporting the pathogenicity of this variant.^19^^,^^20^

This study has a few limitations. We have not performed whole genome sequencing. Therefore, although unlikely, we cannot completely exclude the presence of other nuclear variants which might be involved in the phenotype. We did not find decreased levels of complex V during functional analysis; even in cell clones with homoplasmy, complex V levels were within the normal limits. Although this may seem unexpected, biochemical studies may show normal enzyme levels in LHON mutations.^21^ In *MT-ATP6,* functional disturbances rather than structural changes have been earlier described in other variants.^22^, ^23^, ^24^ Pathologic processes in *MT-ATP6* variants do not seem to affect the production levels of ATP synthase but give rise to unstable complex V resulting in reduced ATP synthesis.^22^ In the light of the aforementioned literature data, additional functional assays, and segregation analysis, we considered the variant m.8969G > A (p.Ser148Asn) the cause of the phenotype in this case.

## 4 Conclusions

*MT-ATP6*-related mitochondrial disease may manifest with LHON. Whole-mitochondrial gene sequencing should be considered in cases of suspected LHON but with negative genetic testing on the common m.3460G > A, m.11778G > A, and m.14484T > C variants.

## Patient consent

The study was approved by the ethical review board of the Academic Medical Center, Amsterdam University Medical Centers. The patient consented to publication of the case in writing.

## Funding

This study was funded by Bartiméus Fonds, the Netherlands, with grant number 1219277. The funder was not involved in the study design; collection, analysis and the interpretation of data or the writing process.

## Authorship

All authors attest that they meet the current ICMJE criteria for Authorship.

## CRediT authorship contribution statement

**Cansu de Muijnck:** Writing – original draft, Visualization, Investigation, Formal analysis, Data curation. **Mary J. van Schooneveld:** Writing – review & editing, Validation, Supervision, Conceptualization. **Astrid S. Plomp:** Writing – review & editing, Validation, Conceptualization. **Richard J. Rodenburg:** Writing – review & editing, Data curation. **Maria M. van Genderen:** Writing – review & editing, Supervision. **Camiel J.F. Boon:** Writing – review & editing, Supervision, Conceptualization.

## Declaration of competing interest

The authors declare that they have no known competing financial interests or personal relationships that could have appeared to influence the work reported in this paper.

The authors have no conflict of interest.
